# Prognostic Impact of Visceral Adipose Tissue Imaging Parameters in Patients with Cholangiocarcinoma after Surgical Resection

**DOI:** 10.3390/ijms25073939

**Published:** 2024-04-01

**Authors:** Jeong Won Lee, Ik Dong Yoo, Sun-pyo Hong, Beodeul Kang, Jung Sun Kim, Yung Kil Kim, Sang Ho Bae, Su Jin Jang, Sang Mi Lee

**Affiliations:** 1 Department of Nuclear Medicine, College of Medicine, Soonchunhyang University Cheonan Hospital, 31 Suncheonhyang 6-gil, Dongnam-gu, Cheonan 31151, Republic of Korea; sads00@naver.com (J.W.L.); 92132@schmc.ac.kr (I.D.Y.); 81907@schmc.ac.kr (S.-p.H.); 2 Division of Medical Oncology, Department of Internal Medicine, CHA Bundang Medical Center, CHA University, 59 Yatap-ro, Bundang-gu, Seongnam 13496, Republic of Koreajungsun@chamc.co.kr (J.S.K.); 3 Department of Surgery, College of Medicine, Soonchunhyang University Cheonan Hospital, 31 Suncheonhyang 6-gil, Dongnam-gu, Cheonan 31151, Republic of Korea; 118553@schmc.ac.kr (Y.K.K.); bestoperator@schmc.ac.kr (S.H.B.); 4 Department of Nuclear Medicine, CHA Bundang Medical Center, CHA University, 59 Yatap-ro, Bundang-gu, Seongnam 13496, Republic of Korea

**Keywords:** adipose tissue, cholangiocarcinoma, obesity, positron emission tomography, prognosis

## Abstract

Visceral adiposity is known to be related to poor prognosis in patients with cholangiocarcinoma; however, the prognostic significance of the qualitative features of adipose tissue in cholangiocarcinoma has yet to be well defined. This study investigated the prognostic impact of adipose tissue imaging parameters reflecting the quantity and qualitative characteristics of subcutaneous (SAT) and visceral (VAT) adipose tissue on recurrence-free survival (RFS) and overall survival (OS) in 94 patients undergoing resection of cholangiocarcinoma. The area, mean computed tomography (CT) attenuation, and mean 2-deoxy-2-[^18^F]fluoro-D-glucose (FDG) uptake of SAT and VAT on positron emission tomography (PET)/CT for staging work-up were measured, and the relationship of these adipose tissue imaging parameters with clinicopathological factors and survival was assessed. TNM stage, histologic grade, lymphovascular invasion, and the size of cholangiocarcinoma showed positive correlations with adipose tissue imaging parameters. Multivariate survival analysis demonstrated that the visceral-to-subcutaneous adipose tissue area ratio (VSR) (*p* = 0.024; hazard ratio, 1.718) and mean FDG uptake of VAT (*p* = 0.033; hazard ratio, 9.781) were significant predictors for RFS, but all of the adipose tissue imaging parameters failed to show statistical significance for predicting OS. In addition to visceral adiposity, FDG uptake of VAT might be a promising prognostic parameter for predicting RFS in patients with cholangiocarcinoma.

## 1. Introduction

Cholangiocarcinoma arises from epithelial cells in the biliary tract, and based on its anatomical location, it is classified into intrahepatic cholangiocarcinoma (when located in the periphery of the second-order bile ducts), perihilar cholangiocarcinoma (when located in the right and left hepatic ducts or at their junction), and distal cholangiocarcinoma (when located in the common bile duct) [1]. Although cholangiocarcinoma is a rare malignant disease, its incidence rate is increasing globally, with the highest incidence seen in Asian countries including Korea [2]. Surgical resection is the sole curative treatment modality for patients with cholangiocarcinoma. However, mainly because of its high recurrence rate and the limited efficacy of chemotherapy, patients who received curative-intent surgical resection have still shown poor prognosis with 5-year overall survival rates between 25 and 40% [3,4]. Therefore, it is imperative to identify prognostic markers that could predict clinical outcomes and refine treatment strategies in patients with cholangiocarcinoma [5].

Alongside other well-known risk factors such as liver fluke infection and cholelithiasis, obesity is emerging as an increasingly important risk factor for the development of cholangiocarcinoma [6]. Furthermore, the body mass index (BMI), which is the most commonly used scale for defining obesity, has been shown to be a significant prognostic factor for predicting clinical outcomes in both intrahepatic and extrahepatic cholangiocarcinoma patients, demonstrating worse survival in patients with a higher BMI [3,4]. Because BMI may be overly broad due to its inability to differentiate adipose tissue mass from other tissue mass in the body, several studies have directly measured areas of subcutaneous (SAT) and visceral adipose tissue (VAT) using computed tomography (CT) images, with such studies finding that adipose tissue mass in the abdomen was significantly associated with survival [7,8,9]. At present, this relationship between adipose tissue mass and cancer progression is mainly attributed to inflammatory processes in the adipose tissue of obese patients [10]. To elaborate, infiltrated immune cells and dysfunctional adipocytes in inflamed adipose tissue secrete pro-inflammatory cytokines and adipokines and cross-talk with cancer cells, which promotes the survival, growth, and metastasis of cancer cells [10,11]. However, the quantitative parameters of adipose tissue, such as BMI and areas of SAT and VAT as measured on imaging studies, are inadequate for directly reflecting inflammatory changes in adipose tissue. Therefore, two imaging parameters, mean computed tomography (CT) attenuation on unenhanced CT images and mean 2-deoxy-2-[^18^F]fluoro-D-glucose (FDG) uptake on positron emission tomography (PET) images, have been proposed to estimate qualitative changes in adipose tissue in imaging studies [12,13,14]. In previous studies, these two imaging parameters measured from SAT and VAT were shown to be correlated with the degree of inflammatory changes in adipose tissue, as well as significantly associated with survival in patients with diverse malignant diseases, including gastric cancer, hepatocellular carcinoma, and colorectal cancer [12,14,15,16,17]. Considering the significant relationship that has been shown to exist between adiposity and the prognosis of cholangiocarcinoma, imaging parameters customized for the qualitative features of adipose tissue could also have significant clinical value for predicting the prognosis of cholangiocarcinoma. However, there has yet to be a study reported on the prognostic significance of CT attenuation and FDG uptake of adipose tissue in patients with cholangiocarcinoma.

Hence, in the present study, we measured both the quantitative and qualitative imaging parameters of SAT and VAT from staging FDG PET/CT images in patients with cholangiocarcinoma treated with surgical resection, and we investigated the prognostic significance of these parameters in predicting recurrence-free survival (RFS) and overall survival (OS).

## 2. Results

### 2.1. Patient Characteristics

The clinicopathological characteristics of the 94 enrolled patients with cholangiocarcinoma are presented in Table 1. The enrolled patients were composed of 66 men (70.2%) and 28 women (29.8%) with a median age of 68 years old. Among the patients, 38 (40.4%) were categorized into the obesity group based on preoperative BMI. Forty-eight patients (51.1%) were treated via complete resection with grossly and microscopically negative margins of resection (R0), while 33 patients (35.1%) and 13 patients (13.8%) received R1 resection (microscopically positive margins of resection) and R2 resection (grossly and microscopically positive margins of resection), respectively. After surgery, 59 patients (62.8%) received adjuvant treatment. The median clinical follow-up duration of the patients was 29.1 months (range, 0.6–113.6 months), and, during the follow-up, 50 patients (53.2%) developed cancer recurrence and 39 patients (41.5%) died.

### 2.2. Correlation Analysis

Table 2 lists the correlation results between the adipose tissue imaging parameters and the clinicopathological characteristics. Among tumor factors, TNM stage, histologic grade, lymphovascular invasion, and tumor size were shown to be correlated with adipose tissue imaging parameters. In particular, for the TNM stage, patients with advanced stages presented with higher values of the visceral-to-subcutaneous adipose tissue area ratio (VSR) (*p* = 0.084) and mean standardized uptake value (SUV) of VAT (VAT SUV) (*p* = 0.061) than those with early stages, and these trends showed borderline significance. In terms of histologic grade, there were significant differences in mean Hounsfield units (HUs) of SAT (SAT HU) (*p* = 0.017), mean HUs of VAT (VAT HU) (*p* = 0.042), and VAT SUV (*p* = 0.008) between the patient groups. Based on the post hoc analysis, patients with moderate and poorly differentiated cancers had significantly higher SAT HU and VAT HU than those with well-differentiated cancers (*p* < 0.05), while patients with poorly differentiated cancers were shown to have significantly higher VAT SUV than those with well-differentiated cancers (*p* < 0.05). Patients who had tumors with lymphovascular invasion had significantly higher SAT HU than those who had tumors with no invasion (*p* = 0.013). Tumor size showed a positive correlation with the visceral adipose index (VAI) with borderline significance (*p* = 0.057). By contrast, tumor classification, perineural invasion, the extent of resection, and serum carbohydrate antigen 19-9 (CA19-9) level did not show a significant correlation with adipose tissue imaging parameters (*p* > 0.10).

Serum C-reactive protein (CRP) was positively correlated with VAI (*p* = 0.090), VSR (*p* = 0.010), and SAT HU (*p* = 0.033). Patients with a recurrence event (n = 50) had significantly higher VAT SUV (*p* = 0.011) and also tended to show higher VAI (*p* = 0.097), VSR (*p* = 0.057), mean SUV of SAT (SAT SUV) (*p* = 0.093), and VAT HU (*p* = 0.052) with borderline statistical significance than those with no recurrence event (n = 44).

The relationship of the adipose tissue imaging parameters with sex and obesity was also evaluated (Table S1). The correlation results revealed significantly higher values of the subcutaneous adipose index (SAI) (*p* < 0.001) in women and significantly higher values of VSR (*p* = 0.001) and SAT HU (*p* = 0.010) in men. Obese patients showed significantly higher values of SAI (*p* < 0.001) and VAI (*p* < 0.001), and significantly lower values of SAT HU (*p* = 0.020) than those with underweight/normal. Moreover, obese patients tended to show higher values of VSR (*p* = 0.090) and lower values of VAT HU (*p* = 0.085) and VAT SUV (*p* = 0.076) with borderline significance.

### 2.3. Survival Analysis for RFS and OS

The prognostic relevance of adipose tissue imaging parameters and clinicopathological factors for predicting RFS and OS was assessed using univariate and multivariate survival analyses. Among adipose tissue imaging parameters, univariate analysis identified VSR and VAT SUV as significant prognostic factors for both RFS and OS, and it identified VAT HU as a significant prognostic factor for RFS only (Table 3; *p* < 0.05). Among clinicopathological factors, TNM stage, extent of resection, histologic grade, perineural invasion, lymphovascular invasion, and serum CRP were significant prognostic factors for predicting both RFS and OS (Table 3; *p* < 0.05).

We further assessed the prognostic significance of adipose tissue imaging parameters and clinicopathological factors for predicting RFS and OS according to the tumor classification using univariate survival analysis. Because of the small number of patients with intrahepatic cholangiocarcinoma, the analysis was performed in patient subgroups of perihilar and distal cholangiocarcinoma (Tables S2 and S3). In the patient subgroup with perihilar cholangiocarcinoma (n = 37), VSR (*p* = 0.008) and VAT SUV (*p* = 0.044) were significant predictors for RFS, whereas none of the adipose tissue imaging parameters showed statistical significance for predicting OS (*p* > 0.05; Table S2). In the patient subgroup with distal cholangiocarcinoma (n = 46), VAT SUV was a significant prognostic factor for predicting both RFS (*p* = 0.001) and OS (*p* = 0.009), and VAT HU was a significant predictor only for RFS (*p* = 0.004; Table S3). Meanwhile, the other adipose tissue imaging parameters including VSR did not reveal statistical significance (*p* > 0.05).

Multivariate survival analysis was performed for adipose tissue imaging parameters that showed statistical significance in the univariate survival analysis (VSR, VAT HU, and VAT SUV). Considering the number of variables compared with the number of events, those three adipose tissue imaging parameters were assessed in separate models including age, sex, obesity, TNM stage, and extent of resection as covariates. The results of the multivariate analysis indicated that VSR (*p* = 0.024; hazard ratio, 1.718 for 1.00 increase; 95% confidence interval [CI], 1.073–2.752) and VAT SUV (*p* = 0.033; hazard ratio, 9.781 for 1.00 increase; 95% CI, 2.137–44.769) remained significant prognostic factors for RFS when controlling for the other factors (Table 4). Contrastingly, all the adipose tissue imaging parameters failed to show statistical significance for predicting OS based on the multivariate analysis (*p* > 0.05).

**Figure 1:**
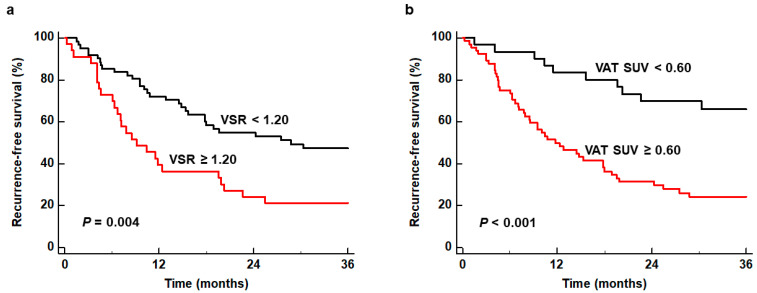
Kaplan–Meier curves of recurrence-free survival according to VSR (**a**) and VAT SUV (**b**).

For estimating cumulative RFS curves, all patients were dichotomized by specific cut-off values of VSR and VAT SUV, which were 1.20 and 0.60, respectively, as determined by receiver operating characteristic (ROC) curve analysis. The ROC curve analysis revealed the area under the ROC curve values of 0.638 (95% CI, 0.532–0.736) for VSR and 0.703 (95% CI, 0.600–0.793) for VAT SUV (Figure S1). Patients with VSR ≥ 1.20 and VAT SUV ≥ 0.60 showed significantly worse RFS than those with VSR < 1.20 and VAT SUV < 0.60 (*p* = 0.004 for VSR and *p* < 0.001 for VAT SUV) (Figure 1). In particular, patients with high VSR and high VAT SUV showed 1-year RFS rates of 39.4% (95% CI, 22.7–56.1%) and 49.8% (95% CI, 37.5–62.1%), respectively, while patients with low VSR and low VAT SUV showed 1-year RFS rates of 72.0% (95% CI, 60.7–83.3%) and 83.3% (95% CI, 70.0–96.6%), respectively.

**Figure 2:**
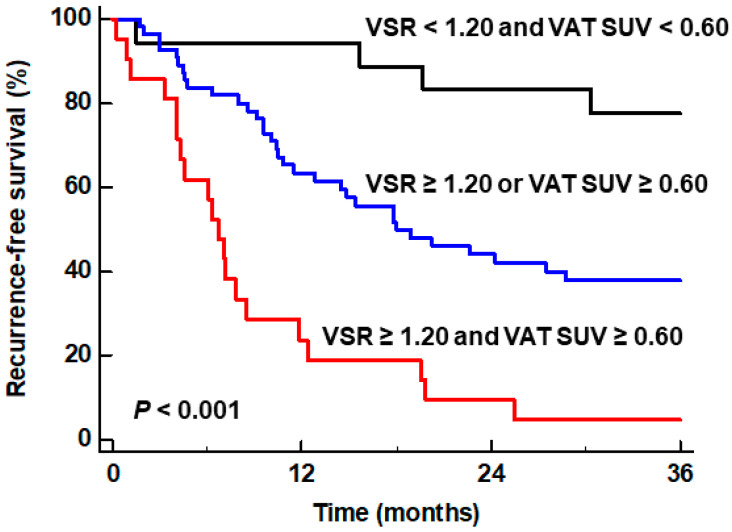
Kaplan–Meier curves of recurrence-free survival of patient subgroups classified by the particular combination of VSR and VAT SUV.

We also investigated whether the combination of both independent predictors could further enhance the prognostic value for predicting RFS. To this end, we classified patients into three subgroups according to the values of VSR and VAT SUV [(1) subgroups of patients with VSR ≥ 1.20 and VAT SUV ≥ 0.60, (2) subgroup of patients with VSR ≥ 1.20 or VAT SUV ≥ 0.60, and (3) subgroup of patients with VSR < 1.20 and VAT SUV < 1.20], and then compared the RFS curves between the groups. The comparisons revealed significant differences in RFS between the three subgroups (*p* < 0.001) (Figure 2). The patient subgroup with VSR ≥ 1.20 and VAT SUV ≥ 0.60 exhibited the worst RFS of all subgroups, showing a 1-year RFS rate of only 23.8% (95% CI, 5.6–42.0%). The patient subgroup with VSR < 1.20 and VAT SUV < 1.20 had the best prognosis with a 1-year RFS rate of 94.4% (95% CI, 83.8–100.0%), while the patient subgroup with VSR ≥ 1.20 or VAT SUV ≥ 0.60 showed a 1-year RFS rate of 63.3% (95% CI, 50.5–76.1%).

## 3. Discussion

In the present study, we investigated the relationship between quantitative and qualitative imaging parameters of SAT and VAT and tumor characteristics and survival in patients with cholangiocarcinoma. In obese patients, adipose tissue undergoes metabolic, functional, and morphological changes, which induce an inflammatory condition that stimulates the development, survival, and progression of various kinds of cancers [18]. Excessive fat accumulation in adipose tissue causes chronic inflammatory processes in the adipose tissue, which induce adipocyte dysfunction and recruit inflammatory cells such as macrophages [10,18]. These dysfunctional adipocytes and infiltrated immune cells in the adipose tissue secrete diverse cytokines and adipokines that provoke a pro-inflammatory microenvironment and remodel the extracellular matrix, thereby providing a favorable microenvironment for cancer growth and metastasis [10,18,19]. Furthermore, studies have shown that adipocytes near the cancer-invasive front are transformed into small-sized cells showing fibroblast-like features, which then release pro-inflammatory cytokines and chemokines, increase glucose metabolism, and provide fatty acids to cancer cells as energy fuel [20,21]. These modified adipocytes are referred to as cancer-associated adipocytes, and they are now considered to have crucial impacts on cancer growth, metastasis, and angiogenesis [21].

This inflammatory process in adipose tissue has been shown to be significantly correlated with CT attenuation and the FDG uptake of adipose tissue in previous studies [15,16,22]. A non-human primate biopsy study demonstrated that increased VAT HU was related to increased extracellular matrix fibrosis and a decreased size of adipocytes in VAT, which is consistent with the findings of inflamed adipose tissue near the cancer-invasive front [22]. Moreover, previous studies investigating the relationship of peritumoral VAT HU and VAT SUV with immunohistochemical findings of peritumoral VAT in patients with gastric cancer and colorectal cancer found that both VAT HU and VAT SUV had significant positive correlations with the degree of interleukin-6 expression and that VAT SUV was further significantly positively correlated with the degree of M2 type macrophage infiltration [15,16]. The results of these previous studies indicated that CT attenuation and FDG uptake of adipose tissue could serve as imaging biomarkers for estimating the degree of inflammatory changes in adipose tissue, suggesting a severe degree of inflammatory changes in adipose tissue in patients with high CT attenuation and FDG uptake [15]. In our study, SAT HU, VAT HU, and VAT SUV were shown to have significant positive correlations with histological grade and lymphovascular invasion of cholangiocarcinoma. Furthermore, the TNM stage had a borderline significant correlation with VSR and VAT SUV, while tumor size also had a borderline significant correlation with VAI. Altogether, these results implied that the degrees of inflammatory changes in SAT and VAT as well as the quantity of VAT were associated with tumor stage and aggressiveness in patients with cholangiocarcinoma. In addition to findings of tumor lesions, preoperative FDG PET/CT could provide valuable information regarding the characteristics of cholangiocarcinoma through adipose tissue imaging parameters.

In previous studies considering cholangiocarcinoma patients, VAI and VSR measured from CT images showed significant associations with disease-free survival and overall survival, specifically by showing poor survival in patients with high VAI and VSR [7,8]. The prognostic significance of CT attenuation and FDG uptake of adipose tissue has yet to be evaluated in patients with cholangiocarcinoma. However, VAT HU and VAT SUV have been shown to be significant prognostic factors for predicting survival in other cancers in the abdominopelvic cavity, including colorectal cancer, gastric cancer, hepatocellular carcinoma, pancreatic cancer, and renal cell carcinoma, all of which grow in adipose tissue-dominant environments [11,12,14,15,23,24]. In the present study, among the adipose tissue imaging parameters, VSR, VAT HU, and VAT SUV were found to be significant predictors for RFS on univariate survival analysis, and VSR and VAT SUV remained as independent predictors for RFS on multivariate analysis after adjusting for age, sex, obesity, TNM stage, and extent of resection. Our results suggest that abdominal adipose tissue distribution and inflammatory change in VAT have significant prognostic importance in cholangiocarcinoma, which could be considered to be imaging evidence showing that VAT features are significantly associated with the recurrence risk of cholangiocarcinoma, similar to other cancers in the abdominopelvic cavity. Other clinicopathological factors that showed statistical significance in the univariate survival analysis, such as pathological tumor stage, extent of resection, histologic grade, perineural invasion, and lymphovascular invasion, can only be evaluated after surgery through histopathological analysis. On the contrary, VSR and VAT SUV can be easily measured from a single image slice of FDG PET/CT, and they also have the advantage of allowing for clinical outcomes to be predicted before surgery. Currently, several studies have attempted to treat cancers by modulating the adipose tissue microenvironment [18,25]. In future clinical trials, the adipose tissue imaging parameters of FDG PET/CT might be a promising imaging biomarker in identifying optimal candidates and speculating treatment outcomes.

In a previous study, high VAI was significantly associated with poor OS as well as RFS on multivariate survival analysis in patients with cholangiocarcinoma [8]; however, in our study, VSR and VAT SUV demonstrated a significant association only with RFS on multivariate analysis and failed to show statistical significance for predicting OS. Another previous study also revealed that VSR did not have a significant association with OS on multivariate survival analysis [7]. The reasons for these different results between the studies are not precisely identified. They might indicate that adipose tissue imaging parameters had a more significant association with cancer progression rather than overall survival [7,8]. However, since our study and the previous studies were retrospectively performed in relatively small population groups of around 100 patients using different imaging parameters and cut-off values, further validation is demanded.

The results of our study demonstrated that the combination of VSR and VAT SUV could further stratify the risk of recurrence after surgery in patients with cholangiocarcinoma. Among the patient subgroups, the subgroups of patients with VSR ≥ 1.20 and VAT SUV ≥ 0.60, who can be considered as having visceral adiposity with enhanced inflammatory change, revealed the worst RFS, with a markedly lower 1-year RFS rate of only 23.8%. Meanwhile, the subgroup of patients with VSR < 1.20 and VAT SUV < 1.20 exhibited a high 1-year RFS of >94.4%. Hence, these results indicate that intense management strategies along with careful surveillance are needed in cholangiocarcinoma patients who show visceral adiposity and increased FDG uptake of VAT on staging work-up PET/CT images.

The current study should be interpreted in light of several limitations. First, because of the retrospective nature of this study, it might have been subject to selection bias. Our results should thus be validated in future prospective studies with larger populations. Second, although no significant differences in adipose tissue imaging parameters were found according to the anatomical location of cholangiocarcinoma, because different cholangiocarcinoma types have been shown to have different tumor microenvironment, further evaluation of the clinical significance of adipose tissue imaging parameters for each type of cholangiocarcinoma might be necessary [26]. On survival analysis in patient subgroups with perihilar and distal cholangiocarcinoma, different adipose tissue imaging parameters showed statistical significance for predicting survival, which suggests that the prognostic impact of the adipose tissue imaging parameters might be different according to the type of cholangiocarcinoma. However, because of the small number of patients in each subgroup, further research is required. Lastly, additional studies with histopathological evaluation are needed to elucidate the underlying mechanism of the results of the present study.

## 4. Materials and Methods

### 4.1. Study Participants

An electronic medical database in two medical centers (CHA Bundang Medical Center and Soonchunhyang University Cheonan Hospital) was retrospectively reviewed to identify patients who were histopathologically diagnosed with intrahepatic, perihilar, or distal cholangiocarcinoma between January 2013 and December 2021. Among these patients, the present study included those who (1) underwent FDG PET/CT for staging work-up, (2) had no evidence of distant metastatic lesion on staging imaging examinations, and (3) received a surgical resection of tumor lesion. We excluded patients who (1) had a previous history of malignant diseases, (2) were diagnosed with combined hepatocellular-cholangiocarcinoma, gallbladder cancer, or bile duct cancers of pathological types other than cholangiocarcinoma, (3) had inappropriate PET/CT images for measuring SAT and VAT imaging parameters, or (4) were lost to follow-up within 12 months after surgery. Based on these inclusion and exclusion criteria, 94 patients in total were ultimately enrolled in the present study.

All patients underwent preoperative staging examinations composed of physical examinations, blood tests, ultrasonography, contrast-enhanced abdominopelvic CT, liver magnetic resonance imaging, and FDG PET/CT. The patients’ weights and heights, measured before surgery, were used to calculate BMI, and all patients were classified into three groups according to the following Asian standards: underweight group < 20.0 kg/m^2^; normal BMI group 20.0–24.9 kg/m^2^; and obese group ≥ 25.0 kg/m^2^ [27]. Following surgical resection, adjuvant chemotherapy and/or radiotherapy were performed based on the pathological tumor stage and clinical condition of the patient. All patients were regularly followed up with blood tests and contrast-enhanced abdominopelvic CT.

### 4.2. Adipose Tissue Imaging Parameter Measurements

All adipose tissue imaging parameters were measured from non-contrast-enhanced CT and PET images obtained from staging work-up FDG PET/CT using the OsiriX MD 10.0 software (Pixemo, Geneva, Switzerland). FDG PET/CT scans were performed with a Biograph mCT 128 scanner (Siemens Healthineers, Knoxville, TN, USA) in both medical centers. The median interval between FDG PET/CT and surgery was 13 days (range, 1–52 days). After at least 6 h of fasting, the patients were intravenously injected with approximately 4.07 MBq/kg (Soonchunhyang University Cheonan Hospital) or 5.18 MBq/kg (CHA Bundang Medical Center) of FDG 60 min prior to scanning. Initially, a non-contrast-enhanced CT scan was conducted using automated dose modulation with a 5 mm slice thickening, and this was followed by a PET scan that was conducted at 1.5 min per bed position.

**Figure 3:**
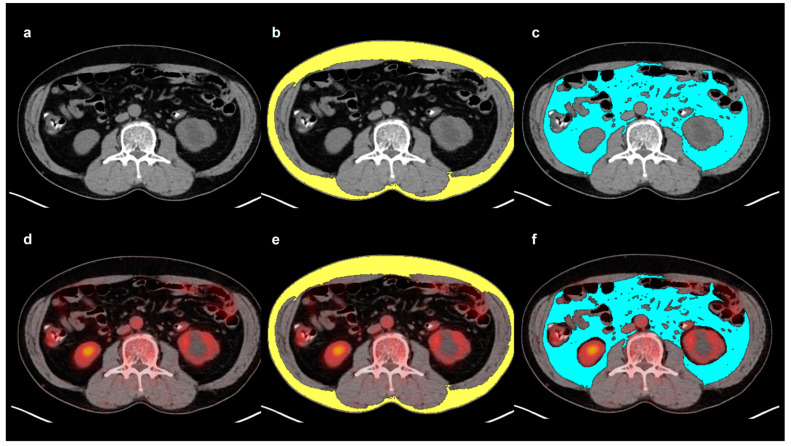
Example measurement of adipose tissue imaging parameters from FDG PET/CT images. Transaxial CT (**a**) and fused PET/CT (**d**) images at the level of the third lumbar vertebra were selected for the measurement. Areas of SAT (**b**) and VAT (**c**) were defined on CT image using HU thresholds of −190 to −30 for SAT (yellow) and −150 to −50 for VAT (blue). The area and the mean HU of both SAT and VAT were measured. Afterward, the SAT and VAT areas on CT images were exported to the corresponding fused PET/CT images (**e**,**f**). After removing the FDG activity of the urine, bowels, and vessels, the mean SUV of SAT and VAT were measured.

All measurements were conducted by the same reader, who was blinded to the clinical information and survival results of the patients. A single slice of the axial CT images was selected at the level of the third lumbar vertebra to determine SAT and VAT areas. SAT and VAT areas (cm^2^) were defined as areas with CT attenuation values ranging from −190 to −30 HU and ranging from −150 to −50 HU, respectively (Figure 3) [7,9]. SAI and VAI were calculated by normalizing the SAT and VAT areas to height squares (cm^2^/m^2^) [27]. The VSR, which reflects the abdominal adipose tissue distribution, was calculated by dividing the VAT area by the SAT area [7]. Moreover, mean CT attenuation values of the SAT and VAT areas were measured and defined as SAT HU and VAT HU, respectively. The SAT and VAT areas on CT images were exported to the corresponding PET images, and the mean SUV of the SAT and VAT areas were also measured, which were defined as SAT SUV and VAT SUV, respectively.

### 4.3. Statistical Analysis

The normality of the distribution of continuous variables, including adipose tissue imaging parameters, tumor size, serum CA19-9 level, and serum CRP level, was assessed using the Shapiro–Wilk test. The Kruskal–Wallis test and Mann–Whitney U test were performed to evaluate differences in the adipose tissue imaging parameters according to the clinicopathological characteristics. For adipose tissue imaging parameters that showed statistically significant differences in the Kruskal–Wallis test, post hoc analysis was conducted using Dunne’s test. Spearman’s correlation coefficients were calculated to assess the relationship of adipose tissue imaging parameters with tumor size, serum CA19-9 level, and serum CRP level. Univariate and multivariate Cox proportional hazards regression analyses were performed to identify the prognostic significance of adipose tissue imaging parameters and clinicopathological factors in predicting RFS and OS. For each variable, a hazard ratio with Wald’s 95% CI was calculated. Survival time was defined as the duration from the day of surgery to the day of diagnosis of cancer recurrence, death, or the last follow-up visit to our medical center. Among the adipose tissue imaging parameters, the parameters that were found to have statistically significant effects on univariate analysis were included in the multivariate analysis for RFS and OS. Each adipose tissue imaging parameter was entered into a separate multivariate survival model after adjusting for age, sex, obesity, TNM stage, and extent of resection. To estimate the cumulative survival curves, optimal cut-off values of adipose tissue parameters were specified through ROC curve analysis. Based on these cut-off values, patients were classified into subgroups, and the Kaplan–Meier method with log-rank test was performed to calculate cumulative RFS curves as well as compare RFS curves between subgroups. MedCalc Statistical Software version 22.016 (MedCalc Software Ltd., Ostend, Belgium) was used for the statistical analysis, and a *p*-value < 0.05 was considered to indicate statistical significance.

## 5. Conclusions

VSR and VAT SUV measured from preoperative FDG PET/CT were found to be independent prognostic factors for predicting RFS in patients with cholangiocarcinoma. Qualitative imaging parameters of SAT and VAT revealed a significant positive correlation with histological grade and lymphovascular invasion of cholangiocarcinoma. Based on the survival analysis, increased VSR and VAT SUV were both associated with an increased risk of cancer recurrence following surgery, and the patient subgroup with both high VSR and VAT SUV showed the worst RFS. VSR and VAT might thus be promising prognostic factors in cholangiocarcinoma patients treated with surgery; however, further studies are needed to validate the results of the current study.

## Figures and Tables

**Table 1:** Clinicopathological characteristics of the patients (*n* = 94).

Factors		Number of Patients (%)
Age (years)		68 (36–81) *
Sex	Men	66 (70.2%)
	Women	28 (29.8%)
Body mass index (kg/m^2^)		24.1 (18.0–32.3) *
Obesity	Obese	38 (40.4%)
	Underweight/normal	56 (59.6%)
Blood tests	CA19-9 (U/mL)	103.0 (0.8–13,328.0) *
	CRP (mg/dL)	4.03 (0.12–1005.10) *
Tumor classification	Intrahepatic	11 (11.7%)
	Perihilar	37 (39.4%)
	Distal	46 (48.9%)
T stage	T1	16 (17.0%)
	T2	26 (27.7%)
	T3	29 (30.9%)
	T4	23 (24.5%)
Lymph node metastasis	Absence	69 (73.4%)
	Presence	25 (26.6%)
TNM stage	Stages I–II	43 (45.7%)
	Stages III–IV	51 (54.3%)
Tumor size (cm)		3.0 (1.1–12.0) *
Histological grade	Well	14 (14.9%)
	Moderately	62 (66.0%)
	Poorly	18 (19.1%)
Perineural invasion	Absence	30 (31.9%)
	Presence	64 (68.1%)
Lymphovascular invasion	Absence	61 (64.9%)
	Presence	33 (35.1%)
Extent of resection	R0	48 (51.1%)
	R1	33 (35.1%)
	R2	13 (13.8%)
Adjuvant treatment	No	35 (37.2%)
	Yes	59 (62.8%)

* Median (range). CA19-9, carbohydrate antigen 19-9; CRP, C-reactive protein; R0, complete resection with grossly and microscopically negative margins of resection; R1, grossly negative but microscopically positive margins of resection; R2, grossly and microscopically positive margins of resection.

**Table 2:** Correlation analysis between adipose tissue imaging parameters and clinicopathological factors.

Factors		SAI	VAI	VSR	SAT HU	SAT SUV	VAT HU	VAT SUV
Tumor classification *	Intrahepatic	32.9 (25.5–47.5)	49.1 (30.2–69.9)	1.22 (0.93–2.13)	−91.8 (−98.7–−86.8)	0.38 (0.34–0.41)	−85.1 (−98.0–−80.1)	0.69 (0.54–0.78)
	Perihilar	45.9 (33.3–81.8)	46.5 (31.1–59.8)	0.87 (0.53–1.30)	−97.7 (−107.0–−91.8)	0.39 (0.34–0.46)	−89.7 (−97.6–−80.9)	0.76 (0.61–0.84)
	Distal	44.8 (35.0–61.2)	46.8 (34.6–61.8)	1.04 (0.70–1.42)	−97.1 (−101.8–−91.3)	0.38 (0.33–0.46)	−89.5 (−95.6–−81.6)	0.63 (0.54–0.86)
	*p*-value	0.165	0.896	0.163	0.226	0.781	0.944	0.475
TNM stage *	Stages I–II	44.9 (35.2–61.2)	43.4 (31.7–55.6)	0.90 (0.57–1.26)	−97.5 (−104.4–−94.6)	0.36 (0.32–0.45)	−91.4 (−99.3–−81.7)	0.62 (0.54–0.79)
	Stages III–IV	44.9 (33.0–64.0)	49.6 (34.2–68.5)	1.16 (0.81–1.48)	−96.1 (−102.1–−89.5)	0.39 (0.34–0.46)	−88.0 (−94.0–−81.6)	0.71 (0.59–0.84)
	*p*-value	0.832	0.105	0.084	0.263	0.191	0.196	0.061
Histological grade *	Well	47.1 (31.7–80.2)	55.2 (36.1–70.7)	1.07 (0.70–1.43)	−104.2 (−107.4–−99.7)	0.37 (0.29–0.43)	−96.5 (−101.7–−85.9)	0.55 (0.52–0.76)
	Moderately	44.6 (33.4–57.8)	46.4 (31.5–61.1)	1.02 (0.62–1.29)	−96.5 (−101.8–−89.5)	0.38 (0.34–0.46)	−88.6 (−94.2–−81.6)	0.68 (0.58–0.83)
	Poorly	48.9 (28.3–70.2)	47.2 (34.3–72.0)	1.03 (0.68–1.53)	−95.1 (−102.1–−90.5)	0.39 (0.33–0.48)	−87.2 (−95.3–−77.2)	0.82 (0.66–0.86)
	*p*-value	0.642	0.404	0.825	0.017	0.424	0.042	0.008
Perineural invasion *	Absence	44.9 (33.0–57.7)	49.2 (34.6–63.8)	1.11 (0.61–1.43)	−97.3 (−102.0–−92.3)	0.39 (0.35–0.47)	−91.6 (−97.4–−85.2)	0.60 (0.54–0.82)
	Presence	45.0 (34.0–66.9)	46.5 (31.8–62.3)	0.97 (0.69–1.35)	−97.1 (−103.5–−90.3)	0.38 (0.33–0.44)	−88.2 (−95.5–−81.3)	0.70 (0.59–0.85)
	*p*-value	0.519	0.733	0.615	0.990	0.226	0.326	0.174
Lymphovascular invasion *	Absence	46.0 (35.4–66.5)	47.3 (34.0–63.0)	0.97 (0.61–1.36)	−99.1 (−104.9–−93.2)	0.38 (0.34–0.45)	−89.8 (−98.2–−83.2)	0.65 (0.55–0.82)
	Presence	37.7 (25.6–54.6)	46.4 (31.9–62.8)	1.16 (0.81–1.52)	−93.6 (−97.7–−86.8)	0.38 (0.33–0.46)	−85.8 (−94.1–−78.8)	0.71 (0.63–0.85)
	*p*-value	0.114	0.666	0.209	0.013	0.612	0.104	0.230
Extent of resection *	R0	45.2 (36.7–58.4)	48.8 (34.8–66.3)	1.14 (0.57–1.37)	−99.4 (−103.9–−92.9)	0.37 (0.33–0.46)	−91.2 (−100.1–−83.6)	0.65 (0.56–0.83)
	R1	38.9 (24.6–68.3)	43.4 (31.4–62.0)	0.97 (0.79–1.41)	−95.6 (−100.8–−86.8)	0.39 (0.35–0.44)	−89.7 (−93.6–−81.4)	0.69 (0.59–0.81)
	R2	41.9 (34.5–73.2)	49.1 (35.4–61.5)	0.97 (0.77–1.50)	−96.1 (−103.8–−89.6)	0.38 (0.34–0.42)	−82.1 (−94.1–−77.1)	0.81 (0.67–0.88)
	*p*-value	0.520	0.737	0.951	0.222	0.735	0.102	0.103
Recur event *	No recurrence	45.1 (34.9–65.3)	37.1 (29.6–55.4)	0.91 (0.61–1.19)	−98.3 (−103.9–−95.0)	0.36 (0.32–0.41)	−91.8 (−99.7–−83.6)	0.59 (0.52–0.72)
	Recurrence	44.6 (30.6–60.1)	49.4 (34.9–63.2)	1.16 (0.73–1.52)	−95.5 (−101.9–−89.5)	0.39 (0.34–0.46)	−87.2 (−94.1–−80.9)	0.77 (0.61–0.87)
	*p*-value	0.744	0.097	0.057	0.119	0.093	0.052	0.011
Tumor size	Correlation coefficient	0.125	0.198	0.152	−0.066	0.013	−0.098	0.040
	*p*-value	0.231	0.057	0.147	0.529	0.904	0.351	0.701
Serum CA19−9	Correlation coefficient	−0.015	−0.038	−0.015	0.099	−0.080	0.136	0.122
	*p*-value	0.885	0.714	0.886	0.342	0.445	0.191	0.242
Serum CRP	Correlation coefficient	−0.085	0.176	0.265	0.219	0.030	0.140	0.037
	*p*-value	0.417	0.090	0.010	0.033	0.771	0.179	0.726

* Expressed in median (interquartile range). CA19-9, carbohydrate antigen 19-9; CRP, C-reactive protein; HU, Hounsfield unit; R0, complete resection with grossly and microscopically negative margins of resection; R1, grossly negative but microscopically positive margins of resection; R2, grossly and microscopically positive margins of resection; SAI, subcutaneous adipose index; SAT, subcutaneous adipose tissue; SUV, standardized uptake value; VAI, visceral adipose index; VAT, visceral adipose tissue; VSR, visceral-to-subcutaneous adipose tissue area ratio.

**Table 3:** Univariate analysis of variables for recurrence-free survival and overall survival following surgical resection.

Variables		Recurrence-Free Survival		Overall Survival	
		*p*-Value	Hazard Ratio (95% CI)	*p*-Value	Hazard Ratio (95% CI)
Age	1-year increase	0.940	0.999 (0.971–1.027)	0.093	1.035 (0.994–1.077)
Sex	Women		1.000		1.000
	Men	0.324	0.762 (0.444–1.308)	0.442	0.769 (0.393–1.503)
Obesity	Underweight/normal		1.000		1.000
	Obese	0.507	1.239 (0.499–1.609)	0.053	1.499 (0.989–2.750)
Tumor classification	Intrahepatic		1.000		1.000
	Perihilar	0.216	1.832 (0.703–4.778)	0.060	6.911 (0.923–51.751)
	Distal	0.541	1.346 (0.520–3.488)	0.135	4.645 (0.621–34.748)
TNM stage	Stages I–II		1.000		1.000
	Stages III–IV	0.001	2.417 (1.410–4.143)	0.025	2.143 (1.100–4.176)
Tumor size	1.0 cm increase	0.098	1.100 (0.980–1.231)	0.125	1.115 (0.970–1.282)
Histological grade	Well		1.000		1.000
	Moderately	0.125	1.965 (0.830–4.653)	0.645	1.256 (0.476–3.311)
	Poorly	0.002	4.551 (1.740–11.904)	0.023	3.459 (1.184–10.108)
Perineural invasion	Absence		1.000		1.000
	Presence	0.007	2.355 (1.265–4.382)	0.008	5.970 (2.107–6.913)
Lymphovascular invasion	Absence		1.000		1.000
	Presence	0.033	1.748 (1.047–2.918)	0.006	2.442 (1.300–4.589)
Extent of resection	R0		1.000		1.000
	R1	0.047	1.777 (1.008–3.134)	0.006	2.765 (1.339–5.710)
	R2	<0.001	3.383 (1.666–6.870)	0.003	4.007 (1.615–9.945)
Serum CA19-9	1.0 U/mL increase	0.790	1.000 (0.999–1.000)	0.438	1.000 (0.999–1.000)
Serum CRP	<5.00 mg/dL		1.000		1.000
	≥5.00 mg/dL	0.045	1.683 (1.011–2.802)	0.044	1.934 (1.019–3.670)
SAI	1.0 increase	0.756	1.002 (0.991–1.012)	0.835	1.001 (0.988–1.015)
VAI	1.0 increase	0.067	1.010 (0.999–1.021)	0.239	1.008 (0.995–1.021)
VSR	1.00 increase	0.023	1.502 (1.114–2.225)	0.047	1.855 (1.029–3.501)
SAT HU	1.0 HU increase	0.094	1.020 (0.997–1.044)	0.322	1.014 (0.986–1.044)
SAT SUV	1.00 increase	0.059	11.354 (0.980–64.065)	0.398	3.468 (0.194–62.099)
VAT HU	1.0 HU increase	0.031	1.027 (1.002–1.053)	0.093	1.026 (0.996–1.058)
VAT SUV	1.00 increase	0.001	18.747 (4.871–72.160)	0.003	10.334 (2.181–48.959)

CA19-9, carbohydrate antigen 19-9; CI, confidence interval; CRP, C-reactive protein; HU, Hounsfield unit; R0, complete resection with grossly and microscopically negative margins of resection; R1, grossly negative but microscopically positive margins of resection; R2, grossly and microscopically positive margins of resection; SAI, subcutaneous adipose index; SAT, subcutaneous adipose tissue; SUV, standardized uptake value; VAI, visceral adipose index; VAT, visceral adipose tissue; VSR, visceral-to-subcutaneous adipose tissue area ratio.

**Table 4:** Prognostic significance of VSR, VAT HU, and VAT SUV for predicting recurrence-free survival and overall survival after adjusting for age, sex, obesity, TNM stage, and extent of resection.

Variables	Recurrence-Free Survival		Overall Survival	
	*p*-Values	Hazard Ratio (95% Confidence Interval)	*p*-Values	Hazard Ratio (95% Confidence Interval)
VSR (1.00 increase)	0.024	1.718 (1.073–2.752)	0.124	1.250 (0.823–2.161)
VAT HU (1.0 HU increase)	0.151	1.023 (0.992–1.054)	0.733	1.006 (0.972–1.042)
VAT SUV (1.00 increase)	0.033	9.781 (2.137–44.769)	0.126	3.479 (0.705–17.180)

HU, Hounsfield unit; SUV, standardized uptake value; VAT, visceral adipose tissue; VSR, visceral-to-subcutaneous adipose tissue area ratio.

## Data Availability

The datasets generated during and/or analyzed in the current study are available from the corresponding authors upon reasonable request.

## Supplementary Materials

The following supporting information can be downloaded at: https://www.mdpi.com/article/10.3390/ijms25073939/s1.
