# Supplementary material for: Prognostic Impact of Visceral Adipose Tissue Imaging Parameters in Patients with Cholangiocarcinoma after Surgical Resection
Source: Int J Mol Sci. 2024 Apr 1;25(7):3939. doi: 10.3390/ijms25073939 (PMC11011754; doi:10.3390/ijms25073939)
Supplement: Supplementary file 1 [file ijms-25-03939-s001.zip › ijms-2925039-supplementary.pdf]

Table S1. Correlation analysis between sex, obesity, and adipose tissue imaging parameters.

Factors		SAI	VAI	VSR	SAT HU	SAT SUV	VAT HU	VAT SUV
Sex	Men	38.6 (27.5–47.7)	46.4 (32.0–64.9)	1.18 (0.88–1.49)	-95.5 (-102.2–-87.2)	0.38 (0.33–0.46)	-88.8 (-96.2–-81.5)	0.68 (0.54-0.83)
	Women	74.3 (52.3–87.2)	47.6 (34.9–60.2)	0.68 (0.44–0.95)	-99.9 (-107.9–-93.5)	0.39 (0.35–0.46)	-89.7 (-96.2–-82.9)	0.69 (0.59–0.87)
	P-value	<0.001	0.980	0.001	0.010	0.869	0.914	0.296
Obesity	Obese	56.3 (45.0–80.2)	63.3 (49.6–79.1)	1.14 (0.76–1.54)	-99.7 (-106.2–-94.5)	0.39 (0.33–0.46)	-87.3 (-101.5–-80.7)	0.69 (0.57–0.83)
	Underweight/normal	37.3 (25.7–47.2)	36.5 (25.4–49.1)	0.91 (0.59–1.27)	-95.9 (-100.8–-88.3)	0.38 (0.33–0.42)	-85.1 (-93.0–-77.5)	0.74 (0.60–0.86)
	P-value	<0.001	<0.001	0.090	0.020	0.357	0.085	0.076

*Expressed in median (interquartile range)

HU, Hounsfield units; SAI, subcutaneous adipose index; SAT, subcutaneous adipose tissue; SUV, standardized uptake value; VAI, visceral adipose index; VAT, visceral adipose tissue; VSR, visceral-to-subcutaneous adipose tissue area ratio

Table S2. Univariate analysis for recurrence-free survival and overall survival in patients with perihilar cholangiocarcinoma (n=37).

Variables		Recurrence-free survival		Overall survival	
		P-value	Hazard ratio (95% CI)	P-value	Hazard ratio (95% CI)
Age	1-year increase	0.447	0.975 (0.944–1.008)	0.995	1.000 (0.958–1.044)
Sex	Women		1.000		1.000
	Men	0.533	0.771 (0.340–1.747)	0.849	0.915 (0.368–2.278)
Obesity	Underweight/normal		1.000		1.000
	Obese	0.757	1.135 (0.508–2.539)	0.385	0.636 (0.229–1.767)
TNM stage	Stage I–II		1.000		1.000
	Stage III–IV	0.035	2.716 (1.022–7.219)	0.044	1.491 (1.053–4.155)
Tumor size	1.0 cm increase	0.005	1.491 (1.125–1.975)	0.003	1.857 (1.240–2.779)
Histological grade	Well		1.000		1.000
	Moderately	0.116	3.269 (0.747–14.313)	0.236	3.456 (0.445–26.863)
	Poorly	0.064	4.534 (0.916–22.437)	0.058	7.672 (0.937–62.822)
Perineural invasion	Absence		1.000		1.000
	Presence	0.040	3.158 (1.052–9.484)	0.030	9.561 (1.248–73.266)
Lymphovascular invasion	Absence		1.000		1.000
	Presence	0.387	1.419 (0.642–3.136)	0.394	1.486 (0.597–3.697)
Extent of resection	R0		1.000		1.000
	R1	0.008	3.452 (1.375–8.666)	0.014	4.306 (1.342–13.812)
	R2	0.005	5.976 (1.737–20.562)	0.019	5.366 (1.312–21.950)
Serum CA19-9	1.0 U/mL increase	0.119	1.000 (0.999–1.000)	0.485	1.000 (0.999–1.001)
Serum CRP	<5.00 mg/dL		1.000		1.000
	≥5.00 mg/dL	0.104	1.913 (0.875–4.180)	0.080	2.281 (0.906–5.742)
SAI	1.0 increase	0.675	0.997 (0.983–1.011)	0.797	0.998 (0.982–1.014)
VAI	1.0 increase	0.233	1.010 (0.994–1.027)	0.172	1.014 (0.994–1.034)
VSR	1.00 increase	0.008	1.948 (1.226–4.572)	0.063	2.381 (0.954–5.940)
SAT HU	1.0 HU increase	0.468	1.012 (0.980–1.045)	0.756	1.001 (0.971–1.041)
SAT SUV	1.00 increase	0.097	6.977 (0.891–109.306)	0.603	3.992 (0.034–340.127)
VAT HU	1.0 HU increase	0.162	1.007 (0.990–1.045)	0.167	1.007 (0.984–1.051)
VAT SUV	1.00 increase	0.044	7.836 (1.473–69.210)	0.178	2.730 (0.629–11.841)

CA19-9, carbohydrate antigen 19-9; CI, confidence interval; CRP, C-reactive protein; HU, Hounsfield units; R0, complete resection with grossly and microscopically negative margins of resection; R1, grossly negative but microscopically positive margins of resection; R2, grossly and microscopically positive margins of resection; SAI, subcutaneous adipose index; SAT, subcutaneous adipose tissue; SUV, standardized uptake value; VAI, visceral adipose index; VAT, visceral adipose tissue; VSR, visceral-to-subcutaneous adipose tissue area ratio

Table S3. Univariate analysis for recurrence-free survival and overall survival in patients with distal cholangiocarcinoma (n=46).

Variables		Recurrence-free survival		Overall survival	
		P-value	Hazard ratio (95% CI)	P-value	Hazard ratio (95% CI)
Age	1-year increase	0.024	1.065 (1.008–1.125)	0.012	1.101 (1.021–1.187)
Sex	Women		1.000		1.000
	Men	0.136	0.561 (0.262–1.200)	0.497	0.997 (0.354–2.806)
Obesity	Underweight/normal		1.000		1.000
	Obese	0.119	1.547 (0.987–1.167)	0.033	1.325 (1.116–1.911)
TNM stage	Stage I–II		1.000		1.000
	Stage III–IV	0.017	2.456 (1.175–5.133)	0.003	4.160 (1.623–10.663)
Tumor size	1.0 cm increase	0.061	1.274 (0.988–1.642)	0.050	1.351 (1.000–1.826)
Histological grade	Well		1.000		1.000
	Moderately	0.574	1.367 (0.460–4.059)	0.816	0.872 (0.274–2.772)
	Poorly	0.016	4.647 (1.325–16.299)	0.284	2.167 (0.527–8.917)
Perineural invasion	Absence		1.000		1.000
	Presence	0.014	3.145 (1.264–7.828)	0.007	7.658 (1.743–33.643)
Lymphovascular invasion	Absence		1.000		1.000
	Presence	0.008	2.707 (1.295–5.660)	<0.001	5.982 (2.219–6.126)
Extent of resection	R0		1.000		1.000
	R1	0.662	1.212 (0.513–2.864)	0.156	2.090 (0.754–5.788)
	R2	0.084	2.169 (0.953–5.672)	0.124	2.615 (0.768–8.904)
Serum CA19-9	1.0 U/mL increase	0.680	1.000 (0.999–1.000)	0.471	1.000 (0.999–1.000)
Serum CRP	<5.00 mg/dL		1.000		1.000
	≥5.00 mg/dL	0.561	1.242 (0.598–2.577)	0.314	1.599 (0.641–3.989)
SAI	1.0 increase	0.573	1.005 (0.988–1.023)	0.984	1.000 (0.978–1.022)
VAI	1.0 increase	0.469	1.006 (0.989–1.024)	0.592	1.006 (0.984–1.028)
VSR	1.00 increase	0.173	1.304 (0.931–2.693)	0.062	1.742 (0.987–4.419)
SAT HU	1.0 HU increase	0.104	1.030 (0.994–1.067)	0.159	1.033 (0.988–1.080)
SAT SUV	1.00 increase	0.097	12.870 (0.628–263.655)	0.639	2.470 (0.057–107.614)
VAT HU	1.0 HU increase	0.004	1.056 (1.018–1.095)	0.057	1.043 (0.999–1.090)
VAT SUV	1.00 increase	0.001	32.627 (5.574–190.985)	0.009	17.917 (2.077–154.546)

CA19-9, carbohydrate antigen 19-9; CI, confidence interval; CRP, C-reactive protein; HU, Hounsfield units; R0, complete resection with grossly and microscopically negative margins of resection; R1, grossly negative but microscopically positive margins of resection; R2, grossly and microscopically positive margins of resection; SAI, subcutaneous adipose index; SAT, subcutaneous adipose tissue; SUV, standardized uptake value; VAI, visceral adipose index; VAT, visceral adipose tissue; VSR, visceral-to-subcutaneous adipose tissue area ratio

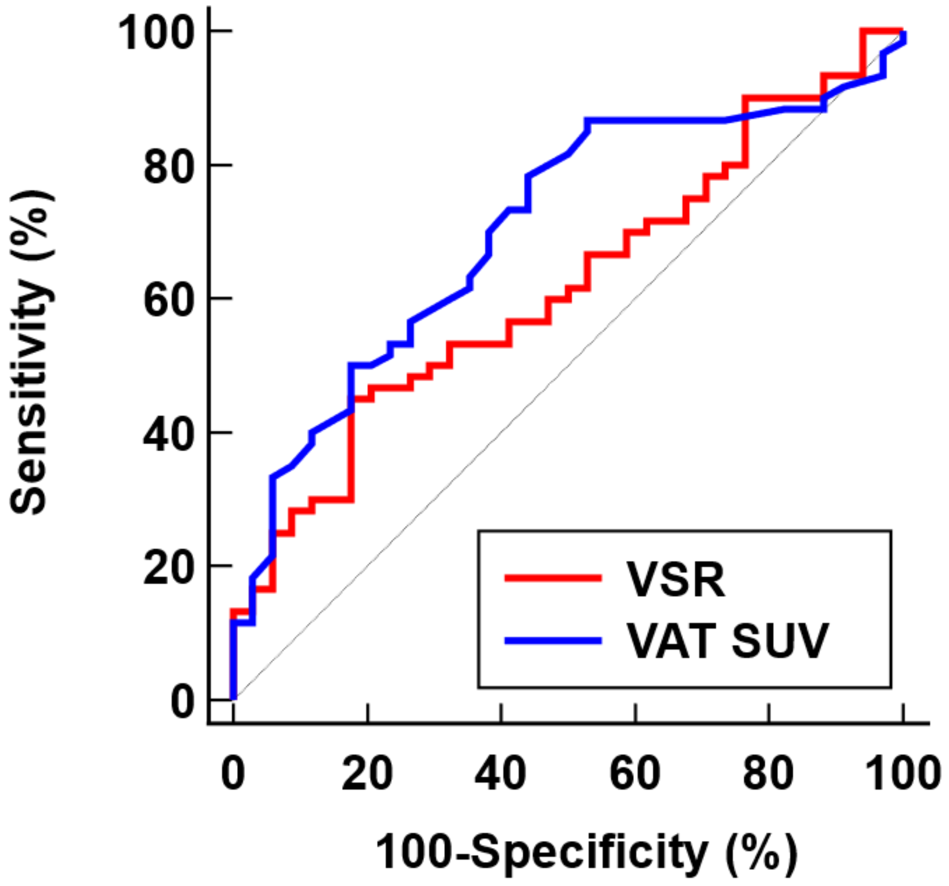

Figure S1. Receiver operating characteristic curves for visceral-to-subcutaneous adipose tissue area ratio (VSR) and mean standardized uptake value of visceral adipose tissue (VAT SUV).
